# Perioperative Positioning in Neurosurgery: A Technical Note on Park Bench Positioning for the Obese Patient Using the “Arrowhead” Technique

**DOI:** 10.7759/cureus.16932

**Published:** 2021-08-06

**Authors:** Dario A Marotta, James Brazdzionis, Brian Fiani, Jason Duong, Jerry Noel, Javed Siddiqi

**Affiliations:** 1 Research, Alabama College of Osteopathic Medicine, Dothan, USA; 2 Department of Neurology, Division of Neuropsychology, University of Alabama, Birmingham, USA; 3 Neurosurgery, Riverside University Health System Medical Center, Moreno Valley, USA; 4 Neurosurgery, Desert Regional Medical Center, Palm Springs, USA; 5 Neurosurgery, Arrowhead Regional Medical Center, Colton, USA

**Keywords:** park bench, posterior fossa, lateral approach, patient positioning, obesity

## Abstract

Complex neurosurgical procedures, such as those traversing the posterior fossa, require optimization of the operative corridor with advanced patient positioning methods. Even seemingly small changes in the location of intracranial mass lesions can require a more dramatic operative trajectory. Modifications of traditional lateral, semi-sitting, and park-bench approaches have been described in the literature to access these lesions; however, technical considerations with respect to enlarged body habitus have yet to be fully explored. Herein, we describe a technique for positioning obese patients in the park bench position, which is referred to as the “Arrowhead technique,” along with a literature review of positional complications and considerations in the setting of obesity.

## Introduction

Proper positioning is the keystone to an effective neurosurgical procedure, both for trajectory to the lesion and to avoid stress complications to non-operated-upon soft tissues. Complex neurosurgical procedures, such as those traversing the posterior fossa, require optimization of the operative corridor with advanced patient positioning methods. Common positioning techniques that provide access to the posterior fossa traditionally include the lateral, prone, and semi-seated positions. Many factors can contribute to the surgeon’s choice of a particular positioning method, to include the surgeon’s training, clinical experience, and personal preferences [1]. Even seemingly small changes in the location of intracranial mass lesions, arteriovenous malformations, and dural fistulas can require a more nuanced trajectory and alterations in patient positioning. As such, modifications of these standard approaches have also emerged in the literature [2-4].

Lateral positioning methods are routinely used and require a coordinated manipulation of the cranium, upper and lower extremities, and torso. The park bench position is a modification of the lateral position that provides enhanced visualization within the posterior fossa [5]. In this modified version lateral position, at least one arm is positioned outside of the operating table, which allows for greater rotation of the shoulders and manipulation of the head and neck. The location of the arms, method of positioning, and stabilization of the arms vary among institutions and surgeons. Patient-specific attributes, particularly body habitus, can complicate positioning efforts. Technical considerations with respect to enlarged body habitus have yet to be fully explored in the literature. Herein, we describe what we refer to as the “Arrowhead technique” for positioning obese patients in the park bench position, along with a literature review of complications related to park bench positioning and surgical considerations in the setting of obesity. For our purposes, “obesity” is defined as per the World Health Organization as a body mass index greater than 30 [6].

## Technical report

**Figure 1:**
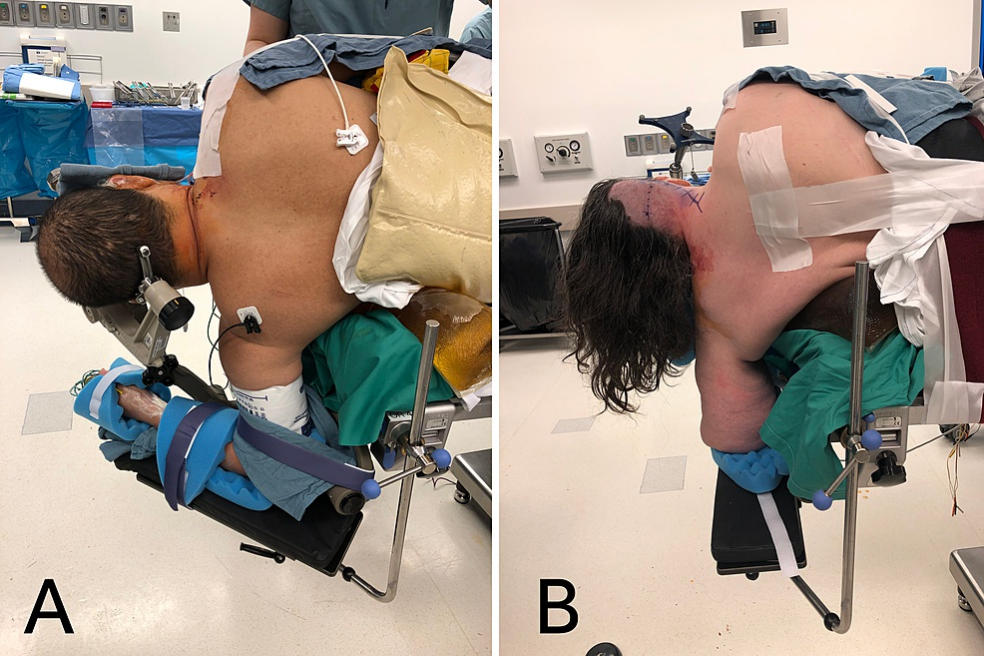
Perioperative images demonstrating proper body and head positioning Proper positioning is shown with shoulders perpendicular to the floor, and head and neck without excessive flexion, extension, or side bending. Multi-position arm boards for the positioning of the lower arm allow for anatomical variations in external rotation of the glenohumeral laxity (A) and shortened humerus length (B).

**Figure 2:**
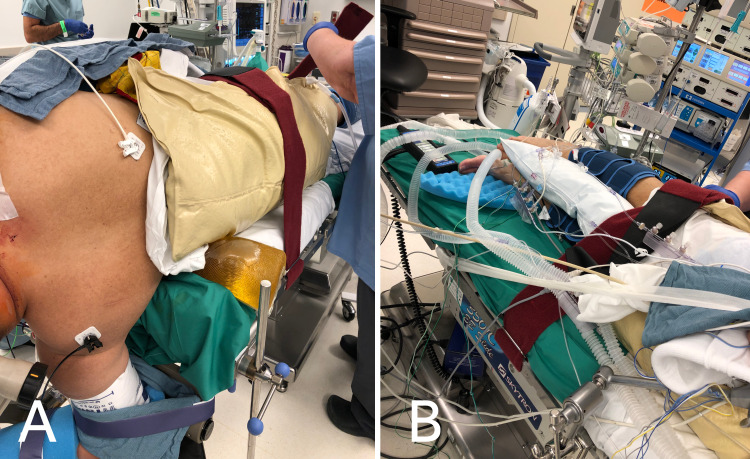
Perioperative images demonstrating proper strap placement Velcro straps are to be placed above the hips (A), above the knees (B), and on the legs. Straps should not be secured over joints, open wounds, or vascular access points. A pillow should be placed between the legs (B) to avoid bony contacts at the level of the ankles and knees.

**Figure 3:**
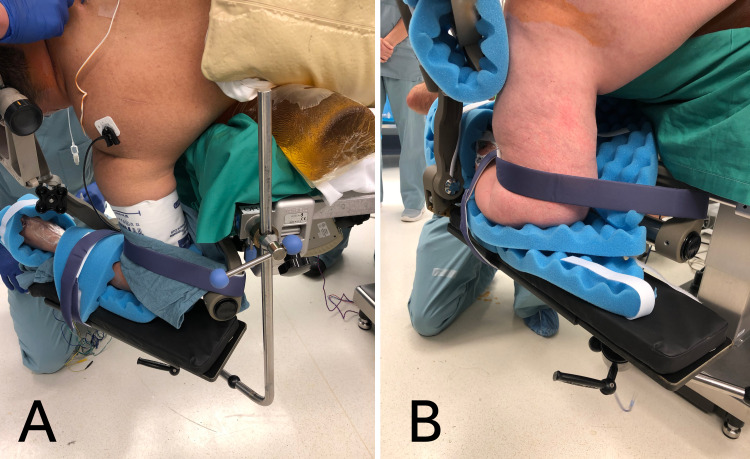
Perioperative images demonstrating proper arm board placement Multi-position arm boards for the positioning of the lower arm allows for anatomical variations in degree of external rotation of the glenohumeral joint (A) and humerus length (B).

**Figure 4:**
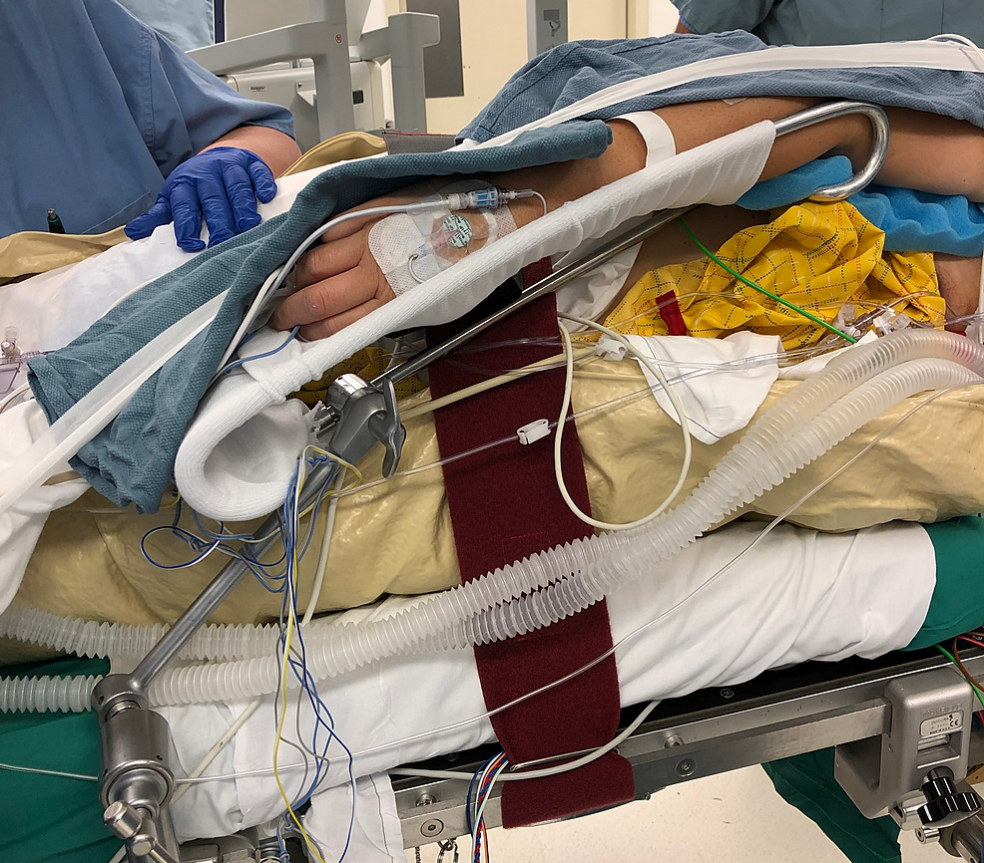
Perioperative image demonstrating proper arm support placement Upper arm support should be placed at the level of the ipsilateral hip in order to open the chest and improve respiration.

**Figure 5:**
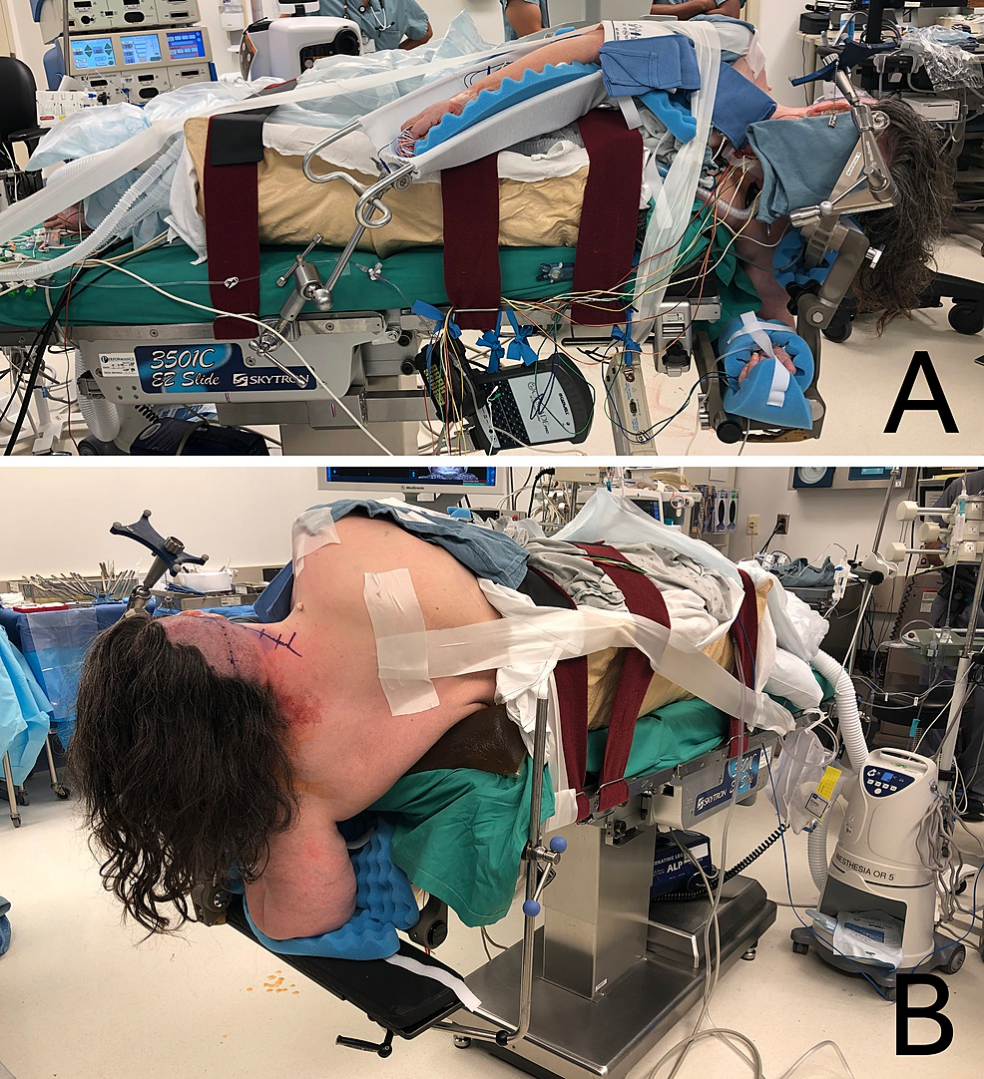
Perioperative images demonstrating proper Arrowhead park bench setup Anterior (A) and posterior (B) aspects of the proper setup of the Arrowhead technique to the park bench position for an obese patient.

Efficient execution of the Arrowhead technique for the park bench positioning for cranial surgery begins with gathering and assembling supplies. For this technique, we utilize a Mayfield skull clamp system, multi-position arm board with bed-rail clamp, Krause-style arm support with properly fit stocking, three Velcro body straps, surgical bean bag positioner, axillary roll, pillow, egg crate foam padding, 3-inch surgical tape, and a draw sheet. Figures 1-5 depict nuances and stages of progress pertaining to the technique.

Preparation

Begin by placing a draw sheet horizontally across the operating table approximately one foot from the head of the table. Situate the surgical bean bag positioner centrally on top of the draw sheet. Affix three Velcro straps to the table rails and allow them to hang freely.

Transfer

After gathering and assembling supplies, the patient is transported to the operating room and transferred to the operating table. Transfer the patient to the operating table supine with the upper edge of the surgical bean bag positioner approximated midway between the patient’s sternal notch and xiphoid process at the level of the nipples. The draw sheet used to transfer the patient onto the bean bag positioner should be retained for future use. Following a successful transfer, the patient undergoes general anesthesia before the table is rotated 180 degrees such that the patient’s feet remain closest to the anesthesia team.

Body and head positioning

Next, a concerted effort is utilized to safely place and secure the patient in the final operative position. Depending on the dimensions and body habitus of the patient, between two and five team members should be present on each side of the patient. The draw sheet under the bean bag positioner is used to slide the patient cephalad until the axilla is at least two fingerbreadths past the edge of the table. Then, the draw sheet between the patient and the bean bag positioner is used to slide the patient laterally toward the side of the surgical approach until the trailing hip is at the center of the table. In a controlled manner, the patient’s leading shoulder is rotated toward the center of the table in a vertical position such that the patient’s coronal plane is perpendicular to the floor. An axillary roll is placed between the caudal edge of the bean bag positioner and the edge of the table before the bean bag positioner is inflated, checked for stability, and verified by a second team member. Figure 1 demonstrates proper body and head positioning.

The Mayfield clamp system is secured to the table with the patient’s head positioned in a manner that optimizes the operative corridor. A pillow is placed between the patient’s legs and knees. Previously affixed Velcro straps are secured at the level of the legs, thighs, and torso. Velcro straps are not to be fastened over vascular access points, joints, or unprotected wounds. Figure 2 demonstrates proper strap placement.

Arm positioning

First, the arm closest to the floor is secured with a multi-position arm board. A clamp is affixed to the side table rail nearest to the patient’s back. The multi-position arm board is inserted into the clamp and positioned above or below the Mayfield clamp depending on the length of the patient’s humerus and laxity of the patient’s glenohumeral joint. Egg crate foam is secured around the patient’s wrist and elbow. Depending on the angle of the arm board, optional Velcro straps can be gently placed above and below the elbow for additional security. After positioning the lower arm, inspect the glenohumeral joint for excess laxity and add padded sub-axillary support as needed. The wrist and elbow joints should contact the arm board without blanching pressure. Figure 3 demonstrates proper arm, torso, and axillary roll positioning.

Next, attach the Krause-style arm support with properly fitted stocking to the table rail on the side facing the patient. Position the arm support parallel to the floor at the height of the patient’s ipsilateral hip with the overarching goal of opening the chest and improving respiration. Egg crate foam should be applied to the patient’s wrists and elbows. Tape can be attached from the patient’s shoulders to the foot of the table to provide gentle caudal traction as needed. Figure 4 demonstrates proper upper arm placement using an upper arm support.

Final inspection

Cables and tubing are organized and secured before vascular access points are verified for functionality and easy access in the case of intraoperative malfunction or emergency. At this point, neuromonitoring leads can be placed and secured while the surgical site is prepared. A final inspection is carried out by at least two team members to ensure the patient is secured and skin is not in direct contact with a solid metal or plastic surface. Figure 5 demonstrates anterior and posterior views of a patient properly positioned using the Arrowhead technique.

## Discussion

In this technical report, we describe the Arrowhead technique for operative positioning of the obese patient in the park bench position for posterior fossa neurosurgical exploration. Positioning of the obese patient exacerbates traditional challenges of complex operative positioning such as lung compliance, airway patency, and pressure-related ulcerations and neuropathies. This technical report was developed to serve as a practical guide for efficiently planning and executing a symphony of maneuvers aimed at optimizing the neurosurgical corridor while minimizing perioperative complications in this susceptible patient population. Table 1 summarizes the 12 steps described within this technical report to function as a perioperative checklist.

**Table 1:** Arrowhead technique guide to patient park bench positioning for the obese patient

Instructions	Equipment Needed	Service
Gather the following supplies prior to patient arrival in the operating room:	Axillary roll, egg crate foam padding, 2-pack x 3, Krause-style arm support with stocking, Mayfield clamp system, multi-position arm board with clamp, pillow, surgical bean bag positioner, tape (3-inch), Velcro straps (3-inch) x 3	Neurosurgery
Place draw sheet and surgical bean bag on the operating table before transferring the patient	Draw sheet, surgical bean bag positioner, transfer board	Neurosurgery
Intubation, vascular access, rotate table with foot of bed near anesthesia	N/A	Neurosurgery, Anesthesia
Affix Mayfield clamp to the patient’s head	Mayfield clamp	Neurosurgery
Position the patient in the following manner: i) Slide patient cephalad such that axillary fold is two fingerbreadths from the edge of the table; ii) Slide patient laterally toward the side of the surgical approach; iii) Rotate ipsilateral shoulder to a vertical position such that the patient is perpendicular to the floor	Draw sheet	Neurosurgery, Anesthesia, Operating Room Staff
Place axillary roll at the edge of the table	Axillary roll	Neurosurgery
Inflate surgical bean bag positioner and stabilize with Velcro straps below the axilla, knee, and pelvis	Surgical bean bag positioner, Velcro straps (3-inch) x 3	Neurosurgery, Operating Room Staff
Position head and clamp Mayfield to the operating table	Mayfield clamp system	Neurosurgery
Consolidate, organize, and secure intravenous and endotracheal tubing. Establish neuromonitoring.	Tape (3-inch)	Anesthesia, Neuromonitoring
Affix arm boards and secure in the following manner: i) Lower: Multi-position arm board above or below Mayfield clamp based on humerus length; ii) Upper: Krause-style arm support with stocking	Multi-position arm board with clamp, Krause-style arm support with stocking	Neurosurgery
Apply padding to upper axilla, elbows, wrists, knees	Egg carton 2-pack x 3, willow	Neurosurgery
Tape arms/shoulder to the foot of the table for traction	Tape (3-inch)	Neurosurgery

Peripheral nerve injuries are fortunately uncommon but can become significant surgical complications in roughly 0.03% of patients undergoing surgery [7]. Unsurprisingly, there is a significant correlation between greater body weight and post-operative paresthesias [4]. Transient ulnar and lateral femoral cutaneous paresthesias are the most common pressure-related neuropathies that tend to resolve by post-operative day 2 [8,9]. Axillary mispositioning is a well-documented source of complications during surgeries utilizing the park bench position. In one case, dramatic depression and fixation of the inferior-most axilla led to loss of radial pulsation, which was quickly identified and corrected [10]. Unnoticed, the potential for limb ischemia during a prolonged surgery speaks to the significance of proper positioning and routine monitoring. We advise positioning the patient’s axilla at least two fingerbreadths from the edge of the table and verifying glenohumeral laxity after inferior arm positioning to avoid similar complications. A distance of at least two fingerbreadths, in our experience, also provides enough support from the torso to mitigate flexion and side bending injuries to the brachial plexus when utilizing rigid head fixation [11]. Finally, a robust axillary roll placed at the edge of the table reduces the potential for pressure-related sequelae associated with lateral thoracic artery compression and subsequent winged scapula [12].

Nearly a quarter of all patients placed in the park bench position develop non-blanchable erythematous Category 1 position-related pressure ulcers [12]. These ulcers are more prevalent in patients undergoing surgeries of greater than 6 hours and those with a core body temperature of more than 100.5°F (38.1°C) at the end of surgery [13]. Small changes in microclimates at the site of pressure points, namely higher skin temperature and average pressure, are predictive of position-related pressure ulcer development. In fact, increases in skin temperature of less than 1°F (0.4°C) and pressures of just 25 mmHg (0.5 pounds per square inch) significantly contribute to ulcer formation. As such, obese patients are at increased risk for ulceration. Removing even a fraction of a pound of pressure from these patients can have a significant impact in preventing post-surgical complications. We utilize egg crate foam because it offers a disposable light-weight pressure relief that can be quickly applied to joints, bony elements, and exposed skin in contact with solid surfaces.

Aside from pressure-related etiologies, airway patency and lung compliance are important positional considerations for obese patients. Obstructive apnea is a common comorbidity, which may complicate intubation and extubating efforts. Physiologically, these patients require greater diaphragmatic excursion to provide the same degree of ventilation. As abdominal and visceral mass accumulates with obesity, intraabdominal pressure increases while functional residual capacity, expiratory reserve volume, and total lung capacity decrease. Fortunately, these patients respond favorably to lateral decubitus surgical positions [14,15]. We elect to rotate the patient perpendicular to the floor, which helps to displace panniculus and improve efficiency of respiration. We also utilize a Krause-style arm support to position the patient’s superior arm at or above the level of the ipsilateral hip to open the chest and improve lung compliance. Finally, it is critical to understand that patient positioning is a team effort, and there must be consensus to the final position between neurosurgery, anesthesia, and nursing; this is especially important as this positioning can take an hour or more of anesthesia time.

## Conclusions

Posterior fossa mass lesions require nuanced positioning techniques to optimize the operative corridor. The park bench position is a routinely utilized method; however, utility in the obese patient has yet to be explored in the literature. Obese patients present anatomical and physiological perioperative challenges. This technical report describes successful positioning of the obese patient at a single institution. Additional research is needed to evaluate and compare similar positioning strategies in this inherently at-risk patient population.
